# Unlocking Therapeutic Potential: Enhanced shRNA Delivery with Tat Peptide in the Human Respiratory Syncytial Virus Treatment

**DOI:** 10.34172/apb.2024.045

**Published:** 2024-05-14

**Authors:** Saeid Amiri Zadeh Fard, Haniyeh Abuei, Abbas Behzad Behbahani, Gholamreza Rafiei Dehbidi, Farahnaz Zare, Maryam Nejabat, Alireza Safarpour, Ali Farhadi

**Affiliations:** ^1^Diagnostic Laboratory Sciences and Technology Research Center, School of Paramedical Sciences, Shiraz University of Medical Sciences, Shiraz, Iran.; ^2^Gastroenterohepatology Research Center, Shiraz University of Medical Sciences, Shiraz, Iran.; ^3^Division of Medical Biotechnology, Department of Medical Laboratory Sciences, School of Paramedical Sciences, Shiraz University of Medical Sciences, Shiraz, Iran.; ^4^Shiraz HIV/AIDS research center, Institute of health, Shiraz University of medical sciences, Shiraz, Iran.

**Keywords:** Fusion gene, Matrix gene, Human respiratory syncytial virus, Real-time PCR, Ribavirin, shRNA, Tat peptide

## Abstract

**Purpose:**

This research investigated the development of short hairpin RNA (shRNA) molecules designed to target specific regions of the human respiratory syncytial virus (HRSV) M and F genes. The study aimed to assess the therapeutic potential of these shRNAs and evaluate the effectiveness of Tat peptide-mediated delivery in enhancing their functionality.

**Methods:**

We acquired isolates from pediatric patients experiencing respiratory illness then cultured in HEp-2 cells. We constructed plasmids expressing shRNAs. Tat peptide as a facilitator for shRNA plasmid delivery was used. The cytotoxicity of ribavirin, shRNA constructs, and control agents was assessed using the MTT assay. The transfection efficiency of Tat peptide-mediated shRNA delivery with that of lipofectamine 3000^TM^ were compared. Finally, real-time PCR was employed to quantify HRSV replication in the treated cells.

**Results:**

Tat peptide-mediated delivery of shRNA plasmids significantly suppressed the expression of the M and F genes of HRSV compared to lipofectamine 3000^TM^. This suppression was evident in both short-term experiments and scenarios involving stable shRNA expression. Furthermore, the combination of ribavirin with shRNA treatment resulted in a substantial reduction in viral load. Notably, the most pronounced antiviral effect was observed when both shRNAs were employed simultaneously.

**Conclusion:**

Our findings suggest that Tat peptide-mediated delivery of shRNA plasmids holds significant potential for achieving stable suppression of HRSV genes. This approach warrants further investigation as a potential gene therapy strategy for HRSV. By demonstrating promising results in vitro, this study highlights the need for future in vivo studies to comprehensively evaluate the therapeutic potential of this approach in a clinical setting.

## Introduction

 Human respiratory syncytial virus (HRSV) is the leading cause of lower respiratory tract infections in infants, significantly compromising their health. This virus primarily damages the lining of small airways in the lungs, causing inflammation, fluid buildup, and cell deterioration, leading to a condition called bronchiolitis. Common symptoms include runny nose, sore throat, cough, headache, fatigue, and fever. Despite its impact, there is currently no vaccine or definitive treatment for HRSV.^1,2^ However, both areas are under active investigation for potential solutions. Ribavirin, an inhaled medication, has been used for treating severe RSV pneumonia in infants and young children, offering some relief for respiratory problems caused by the virus.^3^ Researchers are actively exploring promising alternatives, such as improved antiviral drugs or monoclonal antibodies, to prevent or treat HRSV infection and combat the damage it inflicts.

 Engineered siRNA (small interfering RNA) has emerged as a promising therapeutic approach for various health concerns, including cancer, inflammatory diseases, metabolic disorders, and infectious diseases.^4,5^ Early studies demonstrated the potential of siRNA in fighting viruses like HRSV,^6^ human immunodeficiency virus-1 (HIV-1),^7^ and the influenza virus.^8,9^ Subsequent research suggests that siRNA-mediated silencing of specific HRSV genes can hinder viral progression.^6,10-15^ Building on successful trials in mice using the targeted siRNA, ALN-RSV01, researchers explored its potential to reduce HRSV infections in humans. While a trial with lung transplant recipients showed a decrease in severe cases and bronchiolitis obliterans syndrome worsening, there were no significant differences in viral loads or subjective well-being measures.^16^

 Traditionally, delivering shRNA or siRNA molecules into cells for therapeutic purposes has relied on harsh methods like electron injection,^17^ lentiviral vectors^18^ and lipid-mediated transfection.^19^ While these approaches are effective, they have limitations due to their potential harm to cells.

 For instance, electroporation and lipofectamine are not suitable for use within living organisms (in vivo), and lentiviral vectors, which integrate viral genetic material into the host genome, carry significant safety risks.^20,21^ While non-viral gene delivery methods offer a safer alternative due to their lower cytotoxicity (cell toxicity), they are limited by their inefficiency in delivering the genetic material (low transfection rate). Cell penetrating peptides (CPPs) represent a recent advancement in this field. These short peptides, typically under 30 amino acids long, can effectively cross cell membranes.^22^ Their positive charge allows them to bind to negatively charged nucleic acids (like DNA and siRNA), protecting them from degradation. Studies have shown that CPPs can successfully deliver both DNA and siRNA molecules in both in vitro (laboratory) and in vivo (living organism) settings.^23^ Furthermore, covalently linking cationic peptides (positively charged peptides) with CPPs enhances their ability to deliver genetic material into cells. This combined approach helps overcome the negative charge of DNA, which can hinder its cellular uptake.^24^ In our recent study, we successfully attached a peptide derived from the HIV-1 Tat protein to a vector carrying shRNAs targeting the M and F genes of HRSV.^25^

 Our research aimed to identify new therapeutic molecules for HRSV infection. We designed two novel shRNAs (short hairpin RNAs) targeting the M and F messenger RNA (mRNA) of HRSV. We evaluated the efficacy of these shRNAs delivered by a Tat peptide conjugate, both individually and in combination, in silencing the F and M genes and inhibiting the virus in laboratory experiments (in vitro).

## Materials and Methods

###  Kits and reagents

 Dulbecco’s modified Eagle’s medium (DMEM) was obtained from Thermo Fisher Scientific (USA). Fetal bovine serum (FBS) and penicillin-streptomycin solution (pen/strep 100x) were procured from Gibco, a branch of Life Technologies located in Paisley, United Kingdom. Viral transport medium (VTM) was sourced from Vircell (Granada, Spain). A quantitative real-time PCR (qPCR) kit commercially available VIASURE detection kit (CerTest Biotec, Zaragoza, Spain) was utilized for this purpose. For efficient nucleic acid extraction, the GF1 nucleic acid extraction kit (Vivantis Technologies Co. Malaysia) was chosen. T4 DNA ligase and RevertAid Reverse Transcriptase were obtained from Fermentas (Vilnius, Lithuania). The pUC57 plasmid, a commonly used cloning vector, was purchased from Gene Universal (Hong Kong). cDNA synthesis, a crucial step in gene expression analysis, was performed using a commercially available cDNA synthesis kit from Favorgen Biotech (Taiwan). TRIzol reagent, a versatile reagent for RNA isolation, was acquired from Life Technologies (CA, USA) to facilitate downstream applications, such as qPCR. For efficient cloning of amplified fragments, the pCRII®-TOPO® vector was utilized. This vector was part of the TOPA TA cloning kit procured from Vivantis (Malaysia). A specific probe for qPCR analysis, devoid of the Rox reference dye, was purchased from Amplicon (Herlev, Denmark) to ensure compatibility with the chosen qPCR platform.

###  Preparing virus strain

 To obtain HRSV strains for our research, we collected nasal secretions from hospitalized infants with acute respiratory infections at Namazi Hospital in Shiraz, Iran. This study was approved by the ethics committee at Shiraz University of Medical Sciences (reference number IR. SUMS. REC.1399.1293), and written informed consent was obtained from the parents. Samples were collected in viral transport media (VTM) and stored at 4 °C for up to 24 hours before being frozen at -80°C for further analysis. Real-time PCR using the VIASURE detection kit (CerTest Biotec, Spain) was performed to identify HRSV A and B subgroups before isolation. Confirmed positive HRSV samples were then disrupted and stored in liquid nitrogen for future use.

###  Viral culture

 Specimens of the Human epidermoid carcinoma cells (HEp-2) withstanding destructive infection caused by HRSV were obtained from the Pasteur institute of Iran cell bank (NCBI code C144, ATCC CCL-23). To culture the cells, Dulbecco’s modified eagles’ medium (DMEM) with added 10% fetal bovine serum (FBS) along with 100 U/mL of penicillin and 100 g/mL of streptomycin (all from Gibco, USA) was used. The whole apparatus was kept at 37°C in an environment with 5% CO2.

###  The crafting and development of HRSV M and F gene

**Figure 1:**
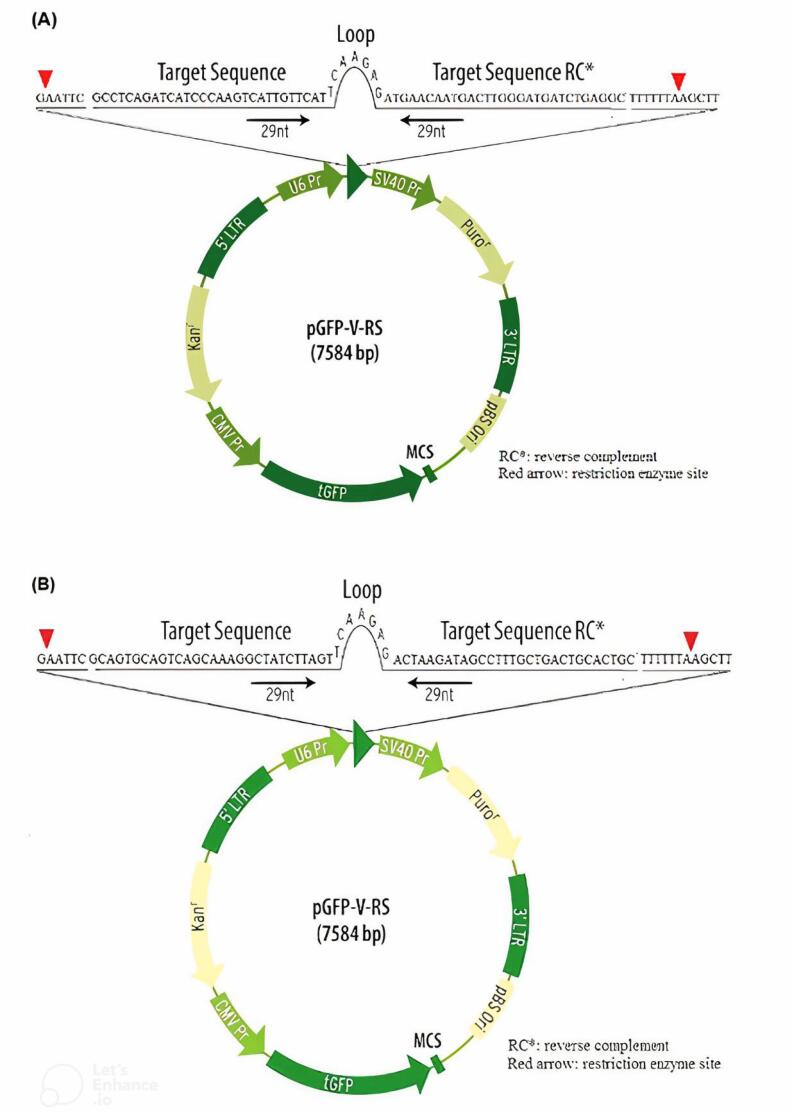
The structure of pGFP-V-RS expression plasmid. (A) contains M-shRNA sequences (B) contains F-shRNA sequences with a particular promoter for the expression of shRNA called U6 and a CMV promoter for GFP expression. The presence of the kanamycin resistance gene in the vector will enable the selection of colonies that have been successfully transformed

 We designed shRNAs targeting the HRSV M and F genes using Biosettia’s online software. This software optimizes shRNA performance, minimizes unintended effects on non-target genes, and ensures sequence compatibility. The designed shRNAs were then evaluated against the human genome and transcriptome databases using the NCBI’s BLAST tool (http://blast.ncbi.nlm.nih.gov/) to ensure specificity. To facilitate identification and later cloning, recognition sequences and restriction enzyme sites (HindIII and EcoRI) were incorporated into the shRNA sequences. A negative control shRNA plasmid was also created containing a sequence with no known homology to human, mouse, or rat genes. Finally, the designed shRNA sequences were inserted into the pUC57 plasmid vector (as shown in Figure 1, obtained from Gene Universal, Hong Kong).

 First, we prepared E. coli bacteria (DH5-α strain) to accept the plasmids containing the shRNA sequences. Then, we extracted the plasmids from an overnight culture of these E. coli using a commercially available DNA extraction kit (GF1, Vivantis Technologies, Malaysia) following the manufacturer’s instructions.

 Next, we digested both the shRNA-containing plasmids (shRNA/pUC) and a separate plasmid called pGFP-V-RS with specific enzymes (EcoRI and HindIII). This process allowed us to insert the shRNA sequences into the pGFP-V-RS vector, which is designed for mammalian cell expression. There is an integrated turbo-GFP element driven by a CMV promoter to readily verify transfection efficiency in this vector.

 The extracted shRNA sequences were then linked to the pGFP-V-RS vector using T4 DNA ligase, an enzyme that joins DNA fragments. Competent *E. coli* (DH5α strain) were transformed with this shRNA-containing vector. We screened several individual bacterial colonies using colony PCR to identify those containing the plasmids with the desired shRNA sequences targeting the HRSV M and F genes. These confirmed recombinant plasmids were then amplified (grown in large quantities) in the same E. coli strain and subsequently purified using a commercially available DNA extraction kit (GF1, Vivantis Technologies, Malaysia) following the manufacturer’s protocol.

###  Tat-Peptide preparation

 Advanced Biomedical (UK) constructed CPP HIV-Tat (49–57) (RKKRRQRRR) with a remarkable level of purity that could reach up to 95%.

###  Combining the peptide and vector

 Our research group recently developed a method that uses non-covalent binding to combine a Tat peptide with a vector containing shRNAs targeting the HRSV M and F genes. In this process, 0.6 micrograms of the Tat peptide were dissolved in a buffer solution (50 μL of HEPES-buffered saline containing 10 mM HEPES, 150 mM NaCl, pH 7.4). Similarly, one microgram of the pGFP-VRS plasmid was suspended in a separate 50-μL solution of 10 mM HEPES buffer (pH 7.4). These solutions were then mixed and incubated for 30 minutes. The amount of Tat peptide used can be adjusted to achieve a desired positive-to-negative charge ratio, depending on the volume of the oligonucleotide solution and the specific target ratio.^26^

 We used a technique called SDS-PAGE to verify the successful attachment (conjugation) of the Tat peptide to the shRNA-containing pGFP-VRS vector. We then compared the efficiency of Tat-mediated transfection to lipofectamine 3000^TM^, a commercially available lipid-based transfection reagent. While no specific reagent exists for shRNA delivery, lipofectamine 3000^TM^ was chosen due to its ability to deliver DNA, which might also be applicable for shRNAs. Following the manufacturer’s instructions (Invitrogen), we prepared a mixture of shRNA/vector complexes with lipofectamine 3000^TM^ in a 1:1 weight ratio. Briefly, 0.5 μL of lipofectamine 3000^TM^ were mixed with25 μL of DMEM (a cell culture medium), followed by the addition of 0.5 μg of shRNA/vector complex diluted in DMEM. This mixture was then incubated for 15 minutes at room temperature.^27^

###  Estimation of zeta potential and size 

 The Tat/Vector complexes were made using a 10 μg/mL concentration of DNA in 10 mM HEPES-buffered saline, as previously mentioned.^28^ The particle size was assessed by dynamic light scattering and zeta potentials calculated by electrophoretic employing the Zetasizer 3000 HS (Malvern Instruments, UK). The assessments were based on an average of three readings.

###  Treatment and transfection

 Hep-2 cells, a type of human epithelial cell line, were grown in 6-well plates at a density of 1.5 million cells per well using low-serum DMEM medium. After 24 hours of incubation at 37 °C, the cells were treated with 100 microliters of the Tat peptide/shRNA/vector complex. The cells were then washed and incubated for an additional 24 to 72 hours in fresh DMEM supplemented with 10% FBS but lacking antibiotics. This process prepared the cells for subsequent assays to evaluate GFP expression. To compare the effectiveness of lipofectamine 3000^TM^ with the Tat peptide method, Hep-2 cells were seeded in 24-well plates at a lower density (100 000 cells per well). These cells were grown for 24 hours in DMEM containing 10% FBS and antibiotics to reach about 80% confluency. Subsequently, each well received a mixture of diluted lipofectamine 3000^TM^ and the shRNA-containing pGFP-VRS plasmid, both of which were prepared separately in serum-free DMEM. This mixture was incubated for 20 minutes before being added to the cells. Similar to the Tat peptide method, the cells were incubated for 24-72 hours to allow for GFP expression.

###  Flow cytometry assay

 We investigated the effectiveness of the Tat peptide in delivering plasmids carrying shRNAs targeting the HRSV matrix and fusion genes into Hep-2 cells. To analyze GFP expression using flow cytometry, the transfected cells were first washed with PBS and treated with a trypsin-EDTA solution to detach them from the culture plate. The cells were then centrifuged to form a pellet, washed again, and resuspended in a cold PBS solution. Finally, fluorescence intensity was measured using a flow cytometer (FACSCalibur) on at least 10 000 cells per sample. The data was further analyzed using FlowJo software (version 10.0).

###  Cytotoxicity studies

 We assessed the cytotoxicity (toxic effects) of shRNAs targeting the HRSV M and F genes, compared to ribavirin, on Hep-2 cells using the MTT assay. 8000 cells were seeded into each well of a 96-well plate and incubated for cell growth. The cells were then exposed to varying concentrations of shRNA/vector complexes, scrambled shRNA (negative control), and ribavirin for 24 hours. Cell viability was measured using a colorimetric assay (MTT assay) with a Tecan Infinite M200 plate reader. Briefly, MTT reagent was added to the cells, and after incubation, the formazan product was solubilized and its absorbance was measured at 570 nm. Higher absorbance values indicate greater cell viability.

 Cell viability (%) = Abs treated cells/Abs untreated cells × 100%.

###  Evaluation of viral load

 The Rotor-Gene Q (Qiagen) platform was used to quantify the amount of HRSV in the sample. It contained 1 μg of cDNA template, Ampliqon real Q Plus 2x Master Mix, 0.4 μM of both primers, and 0.25 μM of the probe (Table 1). The PCR cycle consisted of 15 minutes at 95 °C followed by 45 cycles of 30 seconds at 95 °C and 45 seconds at 52 °C for M gene, 58 °C for F gene, and 30 seconds at 72 °C. Additionally, for every assay performed, several negative controls and standards were included. The quantity of HRSV RNA transcripts (copies/mL) was ascertained based on the standard curve generated. To examine if there is any conflict between the primers and probes needed to identify HRSV within two clinical samples containing high concentrations of both HRSV A and HRSV B were also used (Table 1).

**Table 1:** Sequences of the probes and primers

**Primer name**	**Forward sequence(5´ to 3´)**	**Reverse sequence**	**Product Size(bp)**
M gene	GGAAACATACGTGAACAAGCTTC	GGCACCCATATTGTWAGTGATG	108
Probe	FAM-CACATACACAGCTGCTGTTCAATAC-BHQ		
F gene	TAACCAGCAARGTGTTAGA	GATCATTTGTTATAGGCATATC	246
Probe	FAM-CTATAGTAAATCAACAGAGTTGTCG BHQ		
β-globin	TGTGTTCACTAGCAACCTCAA	CTCACCACCAACTTCATCCA	111
Probe	FAM-CCTGAGGAGAAGTCTGCCGTTACTGCC-BHQ		

Key: W=A, T; R=A, G.

###  Statistical analysis

 The statistical assessment of the figures was conducted via GraphPad Prism version 8.0.0 for Windows, GraphPad Software, San Diego, California USA (https://www.graphpad.com/).

 The MTT trials were overviewed using a one-way ANOVA. For the evaluation of practicality, multiverse analysis and nonlinear regression strategies were adopted. Cited data stands for the average, alongside its standard variation (SD). Academic policy declares any statistical relevance with a *P* value of less than 0.05 to be of significant worth.

## Results and Discussion

 Despite ongoing research, there’s no approved vaccine or effective treatment for HRSV. This study explores using shRNAs to target the virus and prevent replication.

 We designed two shRNAs that silence two crucial HRSV genes (M and F) simultaneously. ShRNAs are attractive as potential therapies due to several advantages: Easy to synthesize, long shelf life, highly specific, can be delivered directly to the respiratory tract (ideal for respiratory viruses), can be combined to target multiple genes or regions, increasing effectiveness and reducing resistance. The growing number of shRNA clinical trials reflects their promising potential.^29,30^

 CPPs have become increasingly popular in recent years for medical and research purposes. These peptides, whether natural or synthetic, can effectively enter cells, allowing for targeted delivery of drugs or other molecules to specific cellular locations. Tat peptides, a type of CPP, are particularly efficient at cellular uptake while maintaining their functionality. However, a drawback of Tat peptides is their susceptibility to breakdown by enzymes due to the presence of positively charged amino acids (arginine and lysine).^31^ A major challenge for CPP-based therapies is ensuring the stability of the CPP itself. The effectiveness of CPPs in delivering drugs can be compromised by their breakdown in bodily fluids or inside cells by enzymes. For instance, studies have shown that Tat peptide, a commonly used CPP, gets degraded by enzymes after entering cells. To address this issue, some researchers have explored using agents like chloroquine to improve CPP stability.^32^ Cationic peptides, which have a positive electrical charge, are often used to attach to plasmids. This attraction works because the positive charges on the peptides interact with the negative charges on the DNA molecules in the plasmids.^33^

###  Combination of Tat-peptide with shRNA/plasmid complexes

 We investigated the properties of complexes formed between Tat peptide, shRNA, and plasmids. We used techniques to measure particle size (dynamic light scattering) and electrical charge (zeta potential). Our results showed that varying the amount of Tat peptide relative to shRNA and plasmid (charge ratio) affected the complex properties (Table 2). Complexes formed with a higher ratio of Tat peptide (10:1) were smaller (around 798 nm) compared to those with a lower ratio (3:1, around 876 nm). The zeta potential of all the complexes is positive. This is because TAT is a cationic peptide, meaning it has a positive charge. Additionally, complexes with a higher Tat ratio had a slightly more positive charge (around 16 mV) compared to those with a lower ratio (around 14 mV). This could be due to the presence of negatively charged DNA in the complex. These findings suggest a connection between the size and charge of the complexes. The experiments were conducted using a buffer solution (10 mM HEPES buffer-saline, pH 7.4) with a set DNA concentration (10 μg/mL). Overall, the TAT peptide can be used to form complexes with DNA. The size and zeta potential of the complexes can be tuned by adjusting the charge ratio of TAT to DNA.

**Table 2:** Average diameter (nm) and zeta potential (mV) of Peptide/DNA complexes prepared in 10 mM HEPES buffer-saline (pH 7.4) using a DNA concentration of 10 μg/mL

**Charge ratio (+/-) **	**Size (nm)**	**Zeta (mV)**
TAT	39 ± 4	19 ± 1.2
TAT/vector 3:1	876 ± 22	14 ± 1.8
TAT/vector 10:1	798 ± 16	16 ± 0.9

The values represent the mean ± SD, n = 3.

###  Cell cytotoxicity assay

**Figure 2:**
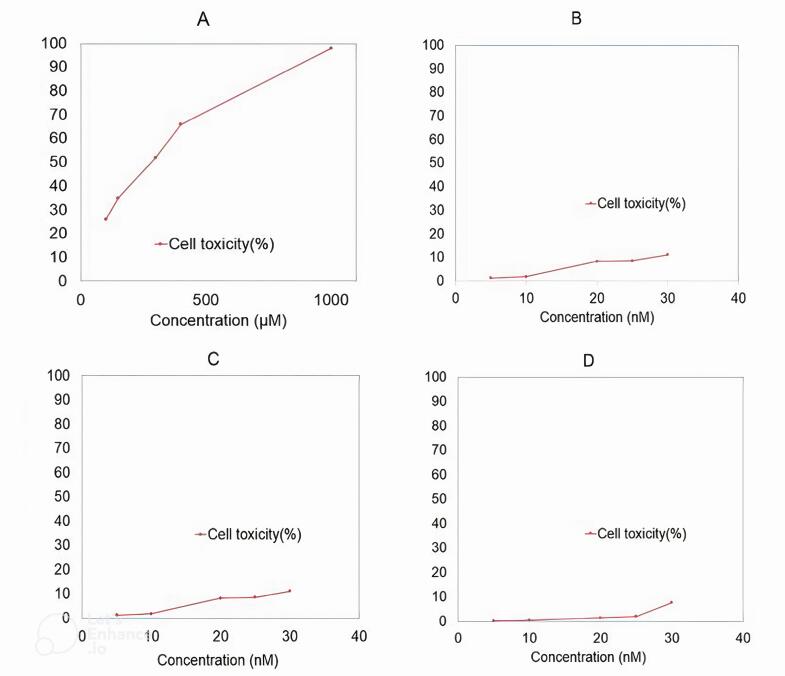
The results of the MTT assay, which determines cell toxicity using different amounts of ribavirin (A), vector containing M-shRNA (B), vector containing F-shRNA (C), and vector containing scramble (D), are presented. For Ribavirin, the TC50 values of 294.6 μM were determined at the 24-hour, 48-hour, and 72-hour post-treatment. The vector containing shRNA and scramble did not exhibit significant toxicity

 We tested the shRNAs for potential unintended effects on healthy cells (cytotoxicity). An assay also compared the cytotoxicity of ribavirin (an antiviral drug) and a control group on Hep-2 cells. The results were promising: the shRNAs caused no significant harm to the cells. Additionally, they didn’t seem to interfere with normal cell growth, as the control cells continued to multiply after transfection. Ribavirin showed the least toxicity at concentrations of 100 and 150 µM (Figure 2A). The shRNAs and the control treatment displayed minimal cytotoxicity at even lower concentrations (Figures 2B and 2C).

###  The pGFP -V-RS –shRNA transfection 

**Figure 3:**
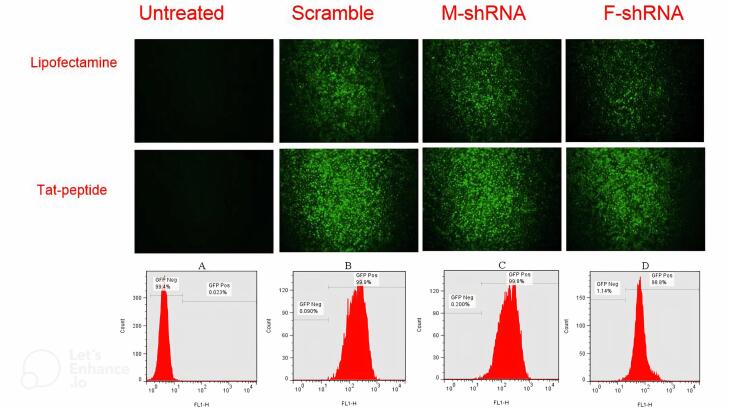
Flow cytometry assay and fluorescent microscopy examination of Hep-2 cells transfected with scramble vector and shRNA/pGFP-V-RS vector harboring HRSV M and F gene compared to untreated cells. A: Untreated cells, B: Hep-2 cells transfected with scramble vector, C: Hep-2 cells transfected with M- shRNA/pGFP-V-RS, D: Hep-2 cells transfected with F- shRNA/pGFP-V-RS

 Analysis of green fluorescent protein (GFP) expression in Hep-2 cells transfected with plasmids containing shRNA targeting the M and F genes revealed successful cellular uptake and translation of the plasmids. Lipofectamine 3000^TM^-mediated transfection of both scrambled and shRNA-expressing plasmids resulted in a significant increase (*P* < 0.0001) in GFP expression compared to untreated cells, reaching approximately 60.2% and 59.5% after 48 hours (Figure 3). Notably, Tat peptide-based transfection achieved a markedly superior outcome, with near-perfect GFP expression (~99.0%) in Hep-2 cells transfected with scrambled or shRNA plasmids at 48 hours. This improvement in transfection efficiency compared to lipofectamine 3000^TM^ was statistically significant (*P* < 0.05).

###  The shRNA suppression of the HRSV proliferation

 Respiratory syncytial virus (RSV) primarily infects epithelial cells in the upper and lower respiratory tracts. To achieve specific silencing of RSV replication, we investigated the use of shRNAs targeting the M and F genes. Subsequently, we employed a lung epithelial cell line to evaluate the efficacy of these shRNAs in inhibiting RSV replication.

 Real-time PCR was used to quantify viral RNA levels and assess the efficacy of shRNAs in suppressing HRSV replication. The assay’s limit of detection (LOD) was established at 1.1 × 10^3^ copies/mL for both HRSV-A and HRSV-B using serial dilutions of a control plasmid.

**Figure 4:**
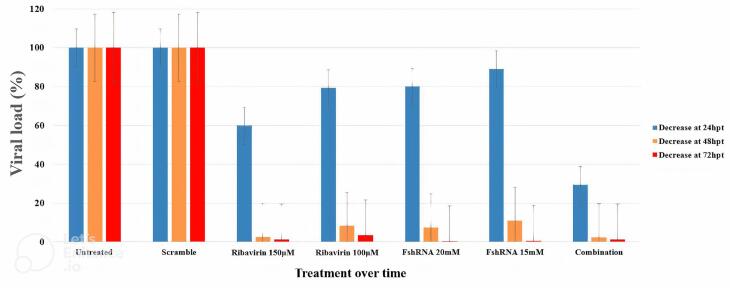
The impact of various concentrations of ribavirin and target shRNA on reducing HRSV viral load over time

 To analyze the effects of shRNAs and ribavirin on HRSV proliferation, we measured viral RNA in the culture medium of Hep-2 cells infected with HRSV. Cells were treated with control vector, shRNA targeting M gene, shRNA targeting F gene, ribavirin alone, combinations of ribavirin with each shRNA, or both shRNAs combined at various concentrations. Viral RNA levels were measured at 24, 48, 72, and 96 hours post-treatment. (Table 3 and Figure 4)

**Table 3:** Tracking the amount of virus present in cells infected with HRSV following administering shRNA and ribavirin as part of a course of treatment over a period of time

**Vector or ribavirin concentrations**	**Viral load (copies/mL)/hours post-treatment**			
	**24**	**48**	**72**	**96**
Untreated control	4.5 × 10^5^	4.2 × 10^6^	5.2 × 10^7^	6.1 × 10^8^
scramble control (20 nM)	4.4 × 10^5^	4.1 × 10^6^	5.0 × 10^7^	6.0 × 10^8^
shRNA targeting HRSV-M gene (10 nM) (5 nM)	3.7 × 10^5^	2.9 × 10^5^	7.5 × 10^4^	9.2 × 10^3^
	4.1 × 10^5^	3.5 × 10^5^	7.9 × 10^4^	10.1 × 10^3^
shRNA targeting HRSV-F gene (10 nM)	3.9 × 10^5^	3.2 × 10^5^	8.0 × 10^4^	10.2 × 10^3^
(5 nM)	4.2 × 10^5^	3.7 × 10^5^	8.3 × 10^4^	10.2 × 10^3^
Ribavirin (150 µM)	3.6 × 10^5^	6.2 × 10^4^	3.2 × 10^4^	9.1 × 10^3^
(100 µM)	4.0 × 10^5^	3.0 × 10^5^	7.5 × 10^4^	9.9 × 10^3^
Ribavirin (150 µM) + shRNA targeting HRSV-M gene (10 nM)	3.4 × 10^5^	4.5 × 10^4^	2.5 × 10^4^	6.9 × 10^3^
Ribavirin (150 µM) + shRNA targeting HRSV-F gene (10 nM)	3.5 × 10^5^	5.1 × 10^4^	3.0 × 10^4^	7.5 × 10^3^
shRNA targeting HRSV-M gene (10 nM) + shRNA targeting HRSV-F gene (10 nM)	3.6 × 10^5^	4.7 × 10^4^	2.6 × 10^4^	7.0 × 10^3^

 Combination therapy with ribavirin and shRNAs targeting the M and F genes of HRSV significantly reduced viral load in Hep-2 cells compared to the control group (*P* = 0.03) (Table 3 and Figure 4). This reduction ranged from 94% to over 99% between 48 and 96 hours post-treatment (hpt). Notably, the combination therapy achieved a more substantial reduction compared to using either shRNA or ribavirin alone.

 Interestingly, both individual shRNAs and the combined shRNA treatment significantly decreased viral load compared to the scrambled control group (*P* < 0.001), with an effect similar to ribavirin treatment. However, unlike ribavirin, shRNAs targeting M and F genes showed a reduction in viral load as early as 24 hpt, with significant effects at 48, 72, and 96 hpt. This suggests that shRNAs may offer a faster-acting therapeutic approach. Overall, our study demonstrates that shRNAs targeting M and F genes effectively inhibit HRSV replication in Hep-2 cells, with combination therapy with ribavirin offering even greater efficacy.

 Our study demonstrates that shRNAs targeting both the M and F genes of HRSV effectively suppress viral replication. This suppression is likely due to a reduction in the entire viral genome, as silencing these critical genes disrupts essential viral processes. Interestingly, silencing the F gene, which is not directly involved in replication, still resulted in a modest reduction in viral RNA. This suggests that targeting F might have additional benefits, such as enhancing the effectiveness of neutralizing antibodies against this protein.

 Previous research using siRNAs has primarily focused on genes crucial for replication (N, P, and M2-2), with consistent success in reducing viral titers. Our findings suggest that targeting non-essential genes, like M, could be a promising strategy. Silencing M might not directly inhibit replication, but it could potentially stimulate the immune response by allowing the accumulation of viral elements, ultimately leading to antibody production. Furthermore, the M protein’s high degree of conservation across RSV strains makes it an attractive target for broad-spectrum shRNA-based therapies.

## Conclusion

 This study investigated a Tat peptide-mediated delivery system for shRNAs targeting the M and F genes of RSV. We employed Tat peptide/shRNA/plasmid complexes and demonstrated a remarkable 99% reduction in viral load after 96 hours using real-time PCR analysis. Significantly, even short-term treatment achieved a 90% reduction, suggesting the potential for rapid intervention.

 The Tat peptide system exceeded lipofectamine 3000^TM^ in silencing M and F genes, leading to enhanced viral replication suppression. The encouraging outcomes of this study elevate the Tat/shRNA system as a putative therapeutic strategy for RSV gene therapy. However, further research is needed to evaluate in vivo efficacy, particularly for sustained silencing using larger nucleic acid constructs. Our findings also suggest the potential application of this approach for treating HRSV infection by suppressing viral replication.

## Competing Interests

 We do not possess any conflicts of interest.

## Ethical Approval

 The study received ethical approval from the Shiraz University of medical sciences research ethics committee, Shiraz, Iran, and reference number IR.SUMS.REC.1399.1293. In addition, all parents of the patients signed the informed consent form after fully understanding the study conditions.
